# Dysregulation of Multiple Facets of Glycogen Metabolism in a Murine Model of Pompe Disease

**DOI:** 10.1371/journal.pone.0056181

**Published:** 2013-02-14

**Authors:** Kristin M. Taylor, Elizabeth Meyers, Michael Phipps, Priya S. Kishnani, Seng H. Cheng, Ronald K. Scheule, Rodney J. Moreland

**Affiliations:** 1 Genzyme, a Sanofi Company, Framingham, Massachusetts, United States of America; 2 Division of Medical Genetics, Department of Pediatrics, Duke University, Durham, North Carolina, United States of America; Wageningen University, The Netherlands

## Abstract

Pompe disease, also known as glycogen storage disease (GSD) type II, is caused by deficiency of lysosomal acid α-glucosidase (GAA). The resulting glycogen accumulation causes a spectrum of disease severity ranging from a rapidly progressive course that is typically fatal by 1 to 2 years of age to a slower progressive course that causes significant morbidity and early mortality in children and adults. The aim of this study is to better understand the biochemical consequences of glycogen accumulation in the Pompe mouse. We evaluated glycogen metabolism in heart, triceps, quadriceps, and liver from wild type and several strains of GAA^−/−^ mice. Unexpectedly, we observed that lysosomal glycogen storage correlated with a robust increase in factors that normally promote glycogen biosynthesis. The GAA^−/−^ mouse strains were found to have elevated glycogen synthase (GS), glycogenin, hexokinase, and glucose-6-phosphate (G-6-P, the allosteric activator of GS). Treating GAA^−/−^ mice with recombinant human GAA (rhGAA) led to a dramatic reduction in the levels of glycogen, GS, glycogenin, and G-6-P. Lysosomal glycogen storage also correlated with a dysregulation of phosphorylase, which normally breaks down cytoplasmic glycogen. Analysis of phosphorylase activity confirmed a previous report that, although phosphorylase protein levels are identical in muscle lysates from wild type and GAA^−/−^ mice, phosphorylase activity is suppressed in the GAA^−/−^ mice in the absence of AMP. This reduction in phosphorylase activity likely exacerbates lysosomal glycogen accumulation. If the dysregulation in glycogen metabolism observed in the mouse model of Pompe disease also occurs in Pompe patients, it may contribute to the observed broad spectrum of disease severity.

## Introduction

Glycogen storage disease type II, also known as Pompe disease and acid maltase deficiency is an autosomal recessive lysosomal storage disorder. It is caused by a deficiency of acid α-glucosidase (GAA; EC 3.2.1.3) an exo-1,4 and 1,6-α-glucosidase that hydrolyzes glycogen to glucose in the lysosome. Deficiency of GAA leads to glycogen accumulation in lysosomes and causes progressive damage to respiratory, cardiac, and skeletal muscle. The disease ranges from a rapidly progressive infantile course that is usually fatal by 1–2 years of age to a more slowly progressive and extremely heterogeneous course that causes significant morbidity and mortality in juveniles and adults [1].

The infantile course of the disease generally correlates with a complete loss of GAA enzyme activity. Unlike the infantile patients, juvenile and adult-onset patients retain partial levels of GAA enzyme activity. In these patients disease severity and age of onset do not correlate with residual enzyme activity [2]. It is therefore likely that secondary factors or modifiers influence the age of onset and severity of the disease in the juvenile and adult-onset patients [1].

One factor that could influence the course of Pompe disease is the level of the glucose transporter 4 (GLUT4). An increase in GLUT4 could potentially result in increased glucose uptake in muscle and consequent increased glycogen accumulation in lysosomes. When muscle biopsies from adult-onset patients were immunostained with an antibody for GLUT4 only the vacuolated glycogen storing muscle fibers showed intense staining for GLUT4 [3]. A similar increase in GLUT4 has been reported in the GAA^−/−^ mouse model for Pompe disease [4]. To our knowledge knockdown of GLUT4 in GAA^−/−^ mice to determine whether elevated GLUT4 causes glycogen accumulation or whether elevated GLUT4 is a consequence of glycogen accumulation has not been reported.

To determine whether other factors in addition to GLUT4 are dysregulated and may contribute to disease severity, we have examined several of the components involved in glycogen biosynthesis and degradation in GAA^−/−^ mice. Unintuitively, many of the steps were dysregulated in a manner that would likely lead to increased glycogen storage. If the dysregulation in glycogen metabolism observed in the mouse model of Pompe disease also occurs in Pompe patients, it may contribute to the observed broad spectrum of disease severity.

## Results

### Glycogen is not reduced in GAA^−/−^ mice lacking S6K1 or S6K2

**Figure 1:**
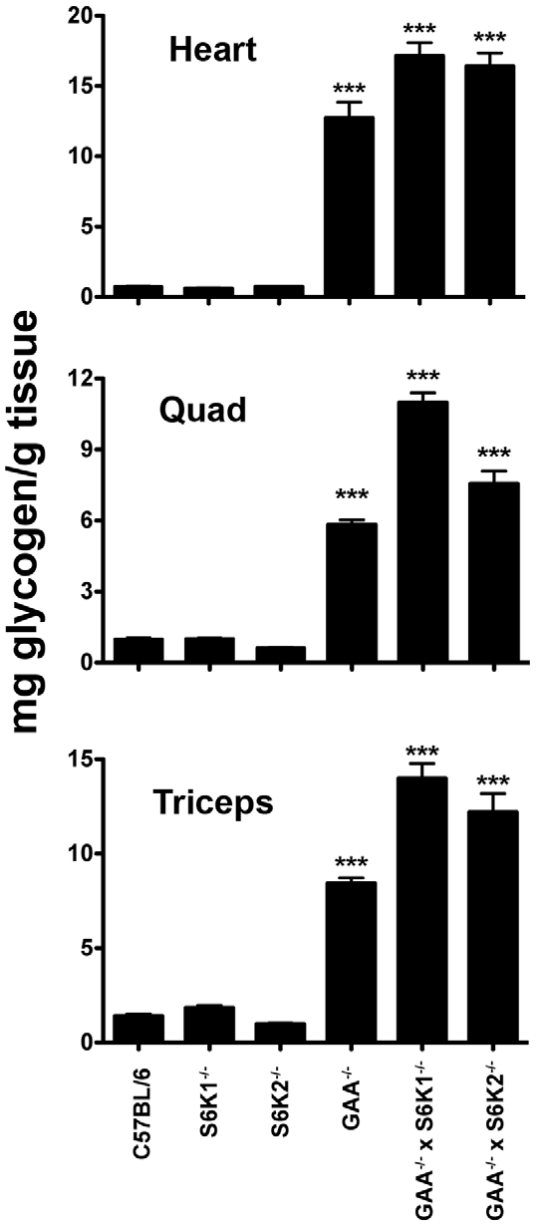
Glycogen is not reduced in GAA^−/−^ mice lacking S6K1 and S6K2. Glycogen was quantified in tissue lysates collected from groups (n = 10) of 3–4 month old mice of the indicated strains. Values shown are means ± SEM. Data was analyzed by one-way ANOVA followed by Newman-Keuls comparing all groups to C57Bl/6. ***P<0.001.

We have previously shown that inhibiting mTORC1 with rapamycin abates glycogen accumulation in skeletal muscle of Pompe mice [5]. However, the therapeutic potential of rapamycin for Pompe disease is limited because rapamycin is also an immunomodulatory agent. It is possible that inhibiting other signaling components in the mTORC1 pathway may prove less immune suppressive and also inhibit glycogen accumulation. As a first step to decipher which components of the mTORC1 pathway might be responsible for rapamycin's ability to inhibit glycogen accumulation, we investigated the S6K1 and S6K2 kinases, which are immediately downstream of and can be phosphorylated by mTORC1 [6]. For this purpose, knockout mice for S6K1 (S6K1^−/−^) and S6K2 (S6K2^−/−^) were crossed to Pompe mice to give the double knockouts (GAA^−/−^ x S6K1^−/−^) and (GAA^−/−^ x S6K2^−/−^). Quantitation of glycogen levels in the heart, quadriceps and triceps from 3–4 month old wild type C57Bl/6, S6K1^−/−^, S6K2^−/−^, GAA^−/−^, GAA^−/−^ x S6K2^−/−^, and GAA^−/−^ x S6K1^−/−^ mice revealed that deleting the genes for S6K1 or S6K2 did not abate the extent of glycogen accumulation noted in Pompe mice (Fig. 1). Histologic examination of muscle sections stained with PAS to visualize glycogen qualitatively confirmed these biochemical results (data not shown). Thus, the S6K1 and S6K2 kinases do not appear to influence muscle glycogen accumulation in the Pompe mouse. Since these double knockouts constitute two additional strains of mice deficient in GAA, they have been used in subsequent studies to evaluate the status of specific components of the glycogen metabolic pathway.

### Phosphorylase is dysregulated in GAA^−/−^ mice

**Figure 2:**
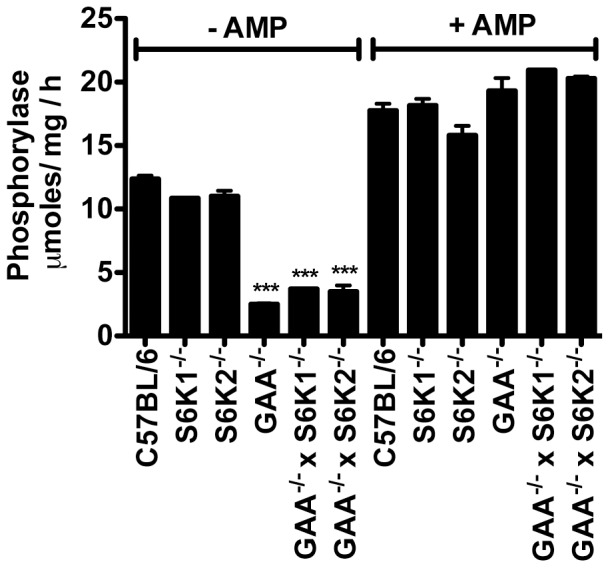
AMP-dependent phosphorylase activity is reduced in GAA^−/−^ mice. Phosphorylase activity was quantified in muscle lysates from triceps collected from groups (n = 5) of 3–4 month old mice of the indicated. Values shown are means ± SEM. Data was analyzed by one-way ANOVA followed by Newman-Keuls comparing all groups to C57Bl/6 in the absence of AMP. ***P<0.001.

Muscle glycogen phosphorylase is one of several enzymes involved in the catabolism of cytoplasmic glycogen. It exists in two forms, the active phosphorylated form (Ph-*a*) and an inactive unphosphorylated form (Ph-*b*). Ph-*b* is only active in the presence of high concentrations of AMP. The ratio of Ph-*a* to Ph-*b* is relevant in Pompe disease because GAA, the other primary enzyme capable of degrading glycogen, is deficient. A situation in which both enzymes that are capable of degrading glycogen are lacking or inactive could result in increased glycogen accumulation. This dynamic appears to exist in GAA^−/−^ mice, as phosphorylase has been reported to be predominantly in the inactive Ph-*b* form in muscle [7]. The generation of additional GAA^−/−^ mouse strains allowed us the opportunity to determine if a reduction in phosphorylase activity is a common feature of GAA deficient mice. Figure 2 shows that in the absence of AMP, phosphorylase activity is greatly reduced in GAA^−/−^, GAA^−/−^ x S6K1^−/−^, and GAA^−/−^ x S6K2^−/−^ mice compared to mice with normal levels of GAA (C57Bl/6, S6K1^−/−^, S6K2^−/−^). The origin of these differences did not appear to be due to differences in absolute phosphorylase levels, since all strains had similar phosphorylase activity when AMP was included in the assay. It has been suggested that elevated glycogen levels may activate the phosphatase that converts Ph-*a* to Ph-*b* [7], but the details of such an activation process are not clear, especially given the different compartmentalization of phosphorylase and glycogen. Thus, together with the above glycogen results (Fig. 1), these results suggest that knockout of GAA leads not only to enhanced glycogen accumulation as expected, but also to a very significant decrease in the basal activity of muscle phosphorylase.

### Glycogen synthase is dysregulated in GAA^−/−^ mice and is normalized by rhGAA treatment

**Figure 3:**
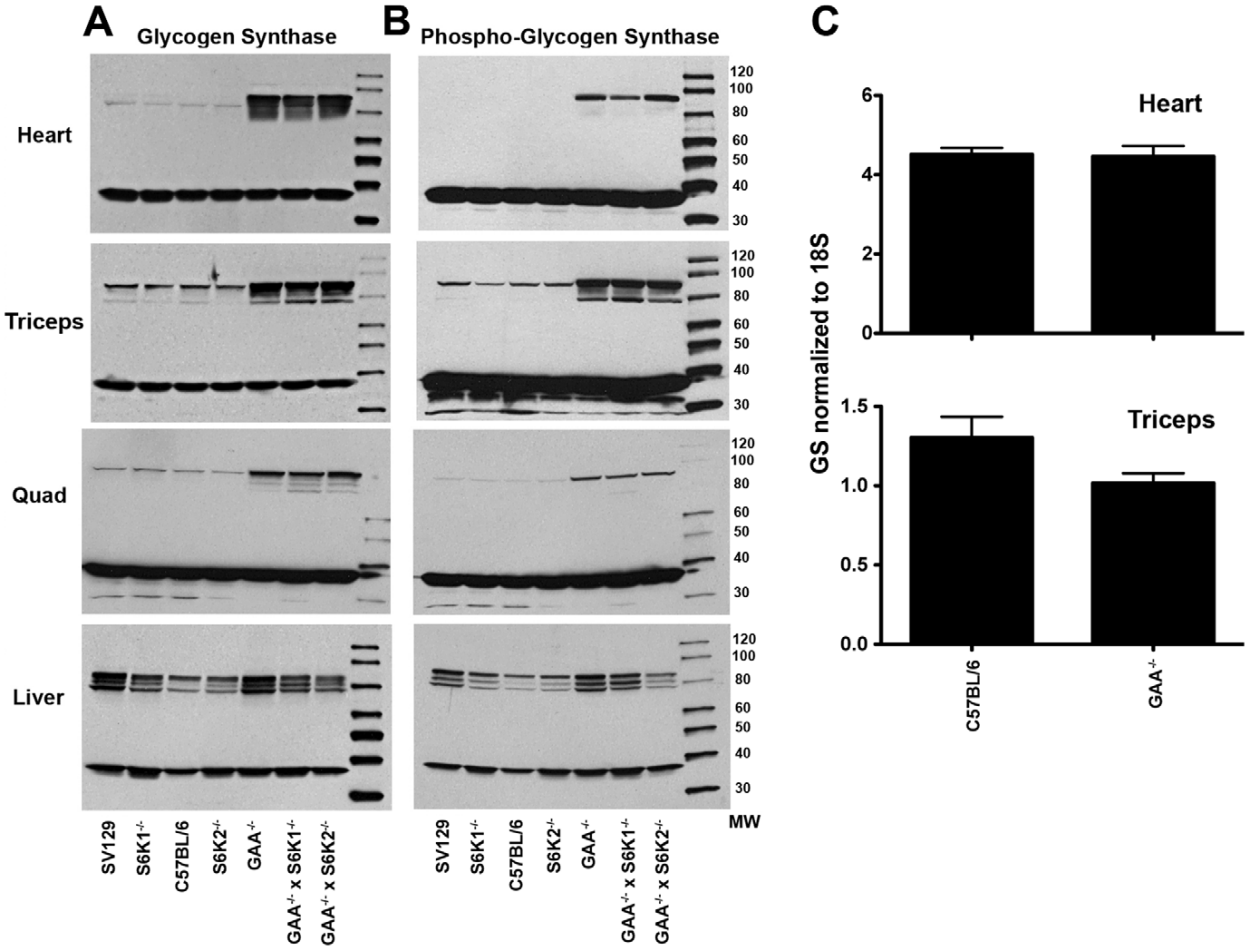
Muscle glycogen synthase protein is elevated in GAA^−/−^ mice. Tissue lysates were prepared for the indicated tissues from each mouse strain. Lysates from 5 mice were pooled and 100 µg of protein from the pooled lysate was analyzed by Western blot analysis. All of the blots were probed with an anti-GAPDH antibody (∼37 kDa in all panels) to verify equal protein loading of all the test samples. (A) An anti-muscle glycogen synthase antibody was used to probe the blots containing samples from the heart, triceps, and quad. An anti-liver glycogen synthase antibody was used to probe the blot containing samples from the liver. (B) An anti-phospho-glycogen synthase (Ser641) antibody was used to probe the blots for phosphorylated GS. (C) Glycogen synthase transcript levels are not elevated in GAA^−/−^ mice compared to wild type animals. RNA was isolated from triceps and heart of C57Bl/6 and GAA^−/−^ mice and probed with qPCR primers as described under “Experimental Procedures.” Values shown are means ± SEM.

Phosphorylase and glycogen synthase activities are normally coordinately regulated in a reciprocal manner. Given the above results with phosphorylase, glycogen synthase (GS) levels and activity in heart, triceps, quadriceps, and liver of SV129 mice together with the strains described in Fig. 1 were surveyed qualitatively by Western blot analysis with antibodies specific to muscle GS and liver GS. Figure 3A shows that compared to the GAA^+/+^ strains, GS levels are elevated in heart, triceps, and quadriceps of all three GAA^−/−^ strains. The most dramatic GS elevation was observed in heart. Interestingly, the levels of liver GS were not altered by knocking out GAA. Figure 3B shows that associated with the increases in absolute GS levels there was a concomitant increase in the amounts of phosphorylated GS in the muscles but not the liver of GAA^−/−^ mouse strains. Glycogen synthase can be inactivated by phosphorylation. However, as we show below, the large increases in GS levels shown here are also accompanied by very significant increases in GS activity.

Figure 3C shows that there were no significant differences in GS mRNA transcript levels in the heart and triceps of wild type C57Bl/6 and GAA^−/−^ mice. There are no differences in GS mRNA levels in quadriceps between wild type C57Bl/6 and GAA^−/−^ mice (data not shown). The increases in GS protein and phosphoprotein noted above (Fig. 3A,B) could be due to differential degradation of the protein in the different mouse strains rather than to its synthesis.

**Figure 4:**
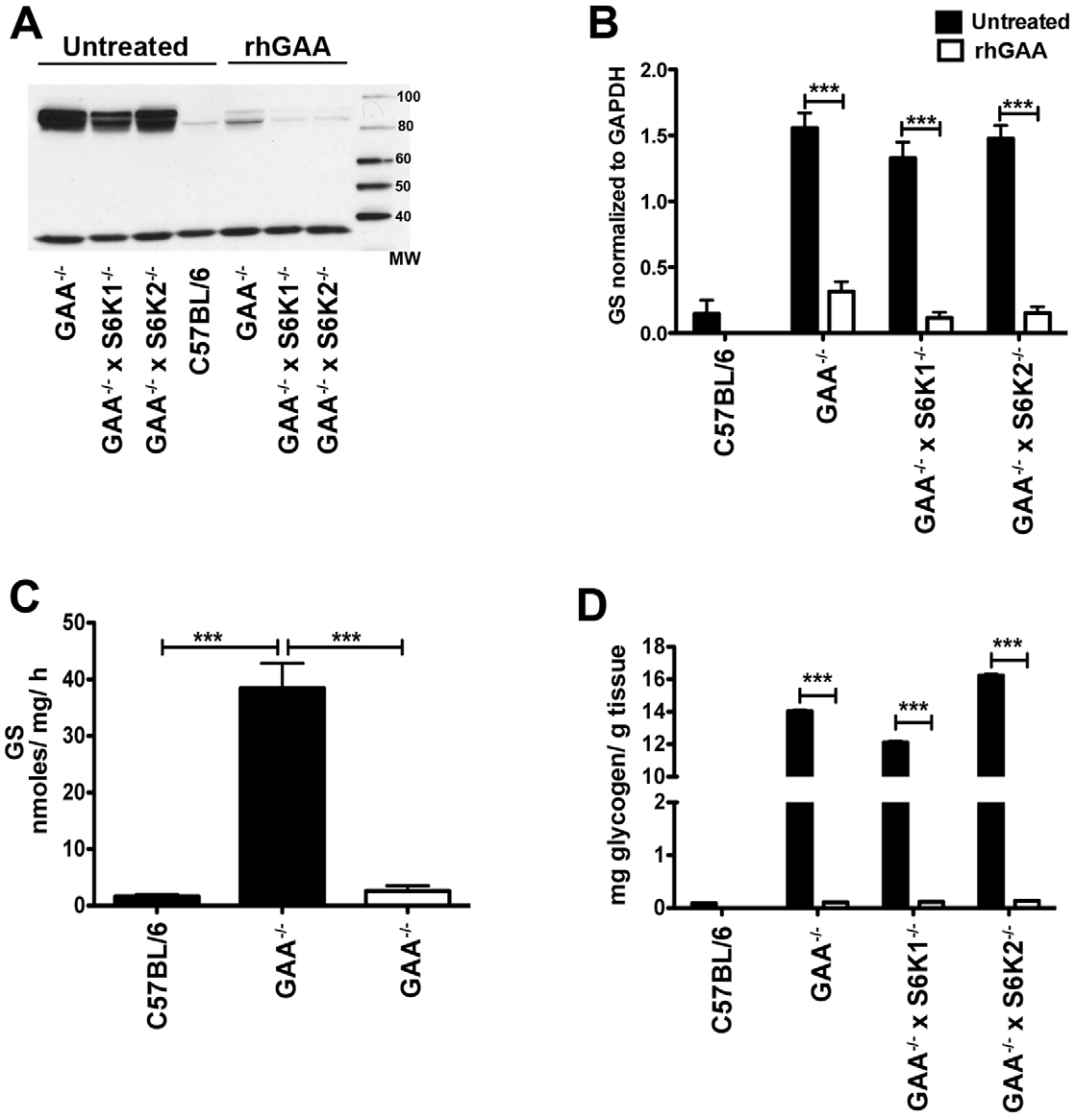
rhGAA treatment normalizes glycogen synthase protein and activity levels in heart of GAA^−/−^ mice. Groups (n = 5) of 3 to 4-month old mice of the indicated strains were dosed with rhGAA (100 mg/kg) weekly for 4 weeks by tail vein injection. Muscle homogenates were prepared, pooled and analyzed by: (A) Western blot using an anti-GS antibody and (B) densitometry analysis of the western blots (with GS normalized to GAPDH)., Samples were also processed for measurements of (C) glycogen synthase activity (D) tissue glycogen levels. Values are means ± SEM. Values are means ± SEM. Data was analyzed by one-way ANOVA followed by Newman-Keuls comparing groups. ***P<0.001.

**Figure 5:**
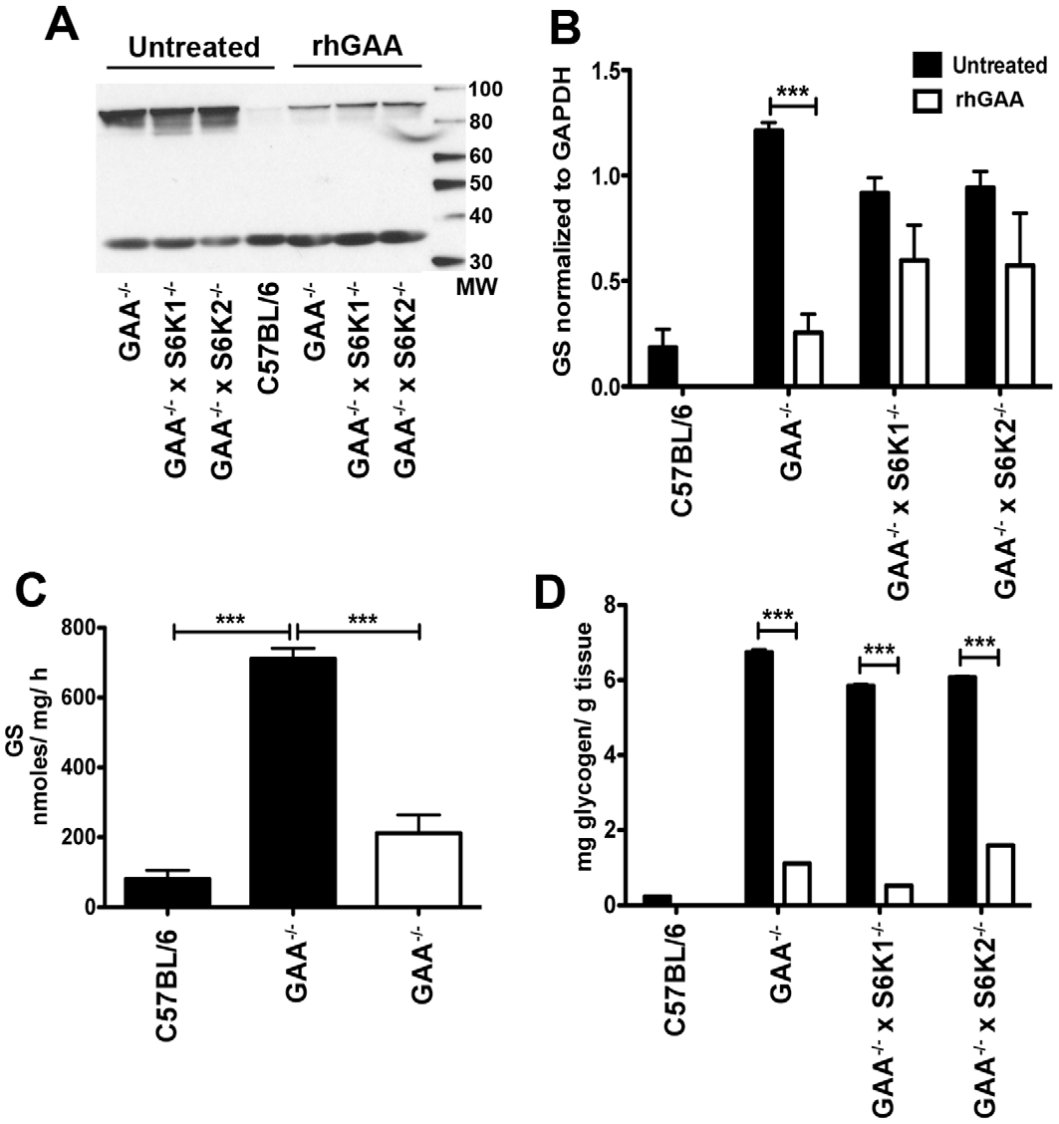
rhGAA treatment reduces glycogen synthase protein and activity levels in triceps of GAA^−/−^ mice. Triceps were analyzed as described in the legend to Figure 4.

Figure 4 gives additional details on GS levels and activity in GAA^−/−^ mouse strains and the effects on cardiac muscle of treating these strains with recombinant human GAA (rhGAA). Four weekly doses of rhGAA (at 100 mg/kg) essentially normalized the hyper-elevated (up to 50-fold) GS levels seen in heart as assessed by Western blot both qualitatively (Fig. 4A) and quantitatively (Fig. 4B). Consistent with this treatment effect on GS levels, treatment also normalized the elevated (∼20 fold) cardiac GS activity (Fig. 4C) and (>100 fold) glycogen levels (Fig. 4D). A similar analysis of triceps treated with rhGAA, showed that GS levels and activity were also dramatically reduced in this muscle, although perhaps not to the same extent as noted in the heart (Figures 5A–C). Consistent with this reduction in GS, glycogen levels in triceps was also greatly reduced, but not completely cleared (Fig. 5D).

Taken together, these results strongly suggest that glycogen synthase levels and activity are dramatically upregulated in the skeletal and cardiac muscles of the Pompe (GAA^−/−^) phenotype, consistent with the coordinate and reciprocal down regulation of phosphorylase shown above (Fig. 2). Especially given the absence of lysosomal acid alpha glucosidase (GAA) in the Pompe mouse, the status of the phosphorylase/synthase balance shown here would be expected to increase glycogen storage further and exacerbate any glycogen-driven pathophysiologic effects.

### G6P and hexokinase activity are elevated in GAA^−/−^ mice and is reduced by rhGAA treatment

**Figure 6:**
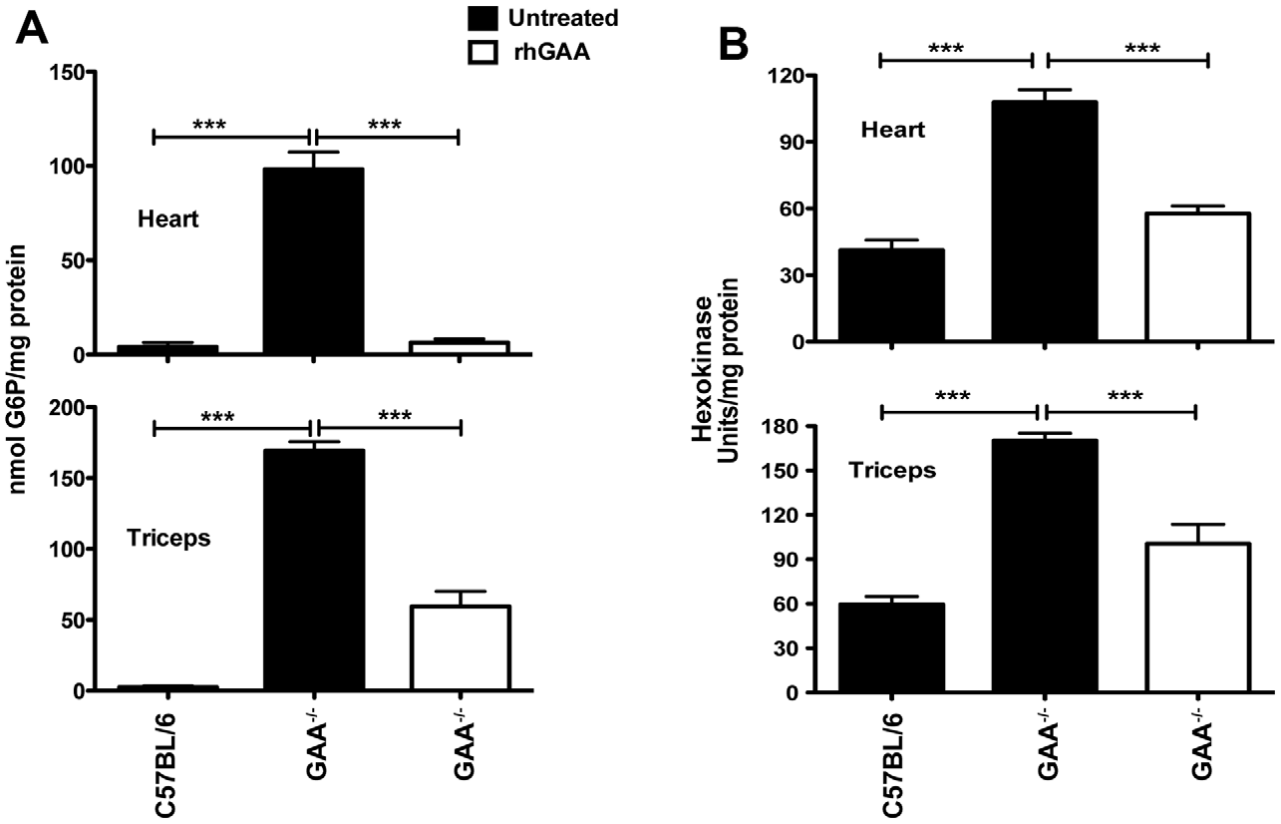
G6P (A) and hexokinase (B) levels are elevated in GAA^−/−^ mice compared to wild type (C57/Bl6) and reduced by rhGAA treatment. GAA^−/−^ mice were dosed with rhGAA as described in the legend to Figure 4. G6P and hexokinase was quantified in heart and triceps homogenates from the strains indicated. Values are means ± SEM. Data was analyzed by one-way ANOVA followed by Newman-Keuls comparing groups. ***P<0.001.

Hexokinase phosphorylates glucose to give G6P. G6P is an allosteric activator of GS and could be contributing to the dramatic GS activity increases in heart and tricep (Figs. 4, 5). G6P levels were compared in heart and triceps from wild type (C57Bl/6) and GAA^−/−^ mice (Fig. 6A). In GAA^−/−^ mice G6P is elevated 20-fold in heart and 60-fold in triceps compared to wild type mice. At these levels, G6P could cause phosphorylated GS to become active and result in greater glycogen biosynthesis. Hexokinase activity is also elevated in GAA^−/−^ mice compared to wild type (C57Bl/6) (Fig. 6B). The high levels of G6P are likely due to increased hexokinase activity in combination with GLUT4, the predominant glucose transporter in the heart and skeletal muscle, which is reportedly elevated in both Pompe patients and GAA^−/−^ mice [3], [4]. Importantly, as with GS, rhGAA treatment reduces levels of G6P and hexokinase in the heart and triceps.

### Glycogenin is dysregulated in GAA^−/−^ mice and normalized by rhGAA treatment

In the presence of UDP-glucose and Mn^2+^, the glycogenin homodimer self-glycosylates and attaches 7–11 glucose residues to a single tyrosine residue [8]. GS then uses this short glucose polymer as a primer to initiate glycogen synthesis. It has been reported that knockdown of glycogenin mRNA levels using short hairpin ribonucleic acids in primary myotubes from GAA^−/−^ mice decreases both cytoplasmic and lysosomal glycogen accumulation [9]. A comparison of muscle glycogenin mRNA or protein levels between wild type mice and GAA^−/−^ mice has not been reported.

**Figure 7:**
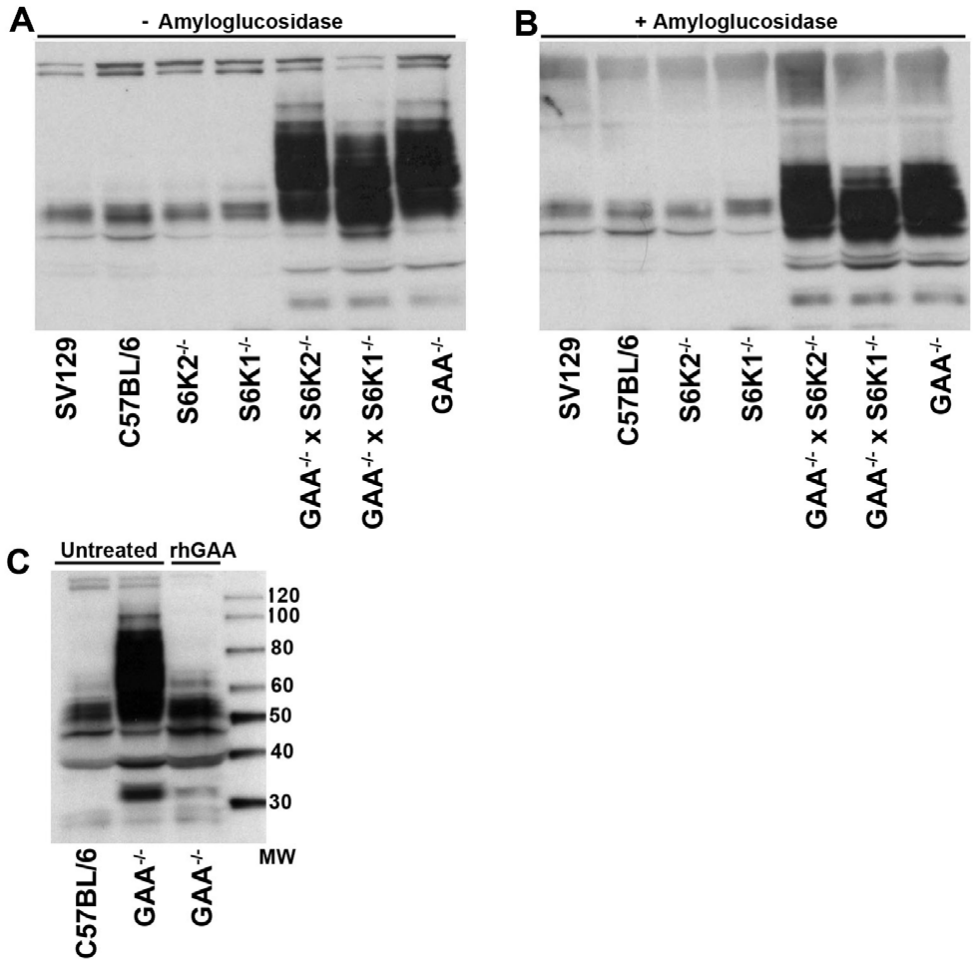
Glycogenin levels are dysregulated in GAA^−/−^ mice and normalized by rhGAA treatment. Homogenates from triceps were prepared for the indicated strains. Lysates from 5 mice for each strain were pooled and 100 µg of protein analyzed by Western blot. A monoclonal antibody to glycogenin was used to probe the blots. (*A*) lysates not treated with amyloglucosidase. (*B)*, treated with amyloglucosidase. (*C*) GAA^−/−^ mice were dosed with rhGAA as described in the legend for Figure 4. Lysates from the strains indicated in this panel were not treated with amyloglucosidase.

Tissue lysates from wild type and GAA^−/−^ mouse triceps muscle were examined by Western blot with a monoclonal antibody specific to muscle glycogenin (Fig. 7, *A–C*). Triceps from GAA^+/+^ strains showed predominant bands ranging between 40–60 kDa in molecular weight. In contrast, triceps from GAA^−/−^ mouse strains revealed a much more intense and diffuse signal from 30–100 kDa, indicating that glycogenin is more heterogeneous and likely upregulated in GAA^−/−^ mice. To ask to what extent this glycogenin heterogeneity was due to attached glycogen, samples were also treated with amyloglucosidase to cleave glycogen covalently attached to glycogenin. Figure 7B shows that this treatment resulted in only a marginal reduction in the heterogeneity of glycogenin suggesting that covalently attached glycogen is not responsible for the observed differences in glycogenin between wild type and GAA^−/−^ mice. Regardless of the origin of this heterogenity, when GAA^−/−^ mice were treated with rhGAA, the molecular form of glycogenin was restored to that of wild type mice, at least as assessed by Western blot analysis (Fig. 7C).

## Discussion

To date, only a few molecular aspects of the glycogen metabolic pathway in Pompe disease have been investigated in detail. For example, it has been reported that GLUT4 is elevated in both Pompe patients and GAA^−/−^ mice [3]. In this report, we confirmed [7] that phosphorylase activity is suppressed in GAA^−/−^ mouse muscle, and further demonstrated that GS, G6P, hexokinase, and glycogenin are all hyper-elevated. The dysregulation observed for GLUT4, GS, G6P, hexokinase, phosphorylase, and glycogenin in combination with a reported increase in autophagy in Pompe disease [10], [11] would appear to comprise an underlying metabolic syndrome. The molecular entities that comprise this metabolic syndrome are all dysregulated in a manner that would facilitate glycogen biosynthesis and ultimately lead to a greater accumulation of lysosomal glycogen.

It is currently unknown whether these dysregulated components cause glycogen accumulation or if their dysregulation is a consequence of the aberrant accumulation of lysosomal glycogen in Pompe disease. It is likely that the observed increases in GS and autophagy cause glycogen accumulation because suppression of either autophagy or GS alone in GAA^−/−^ mice reduced glycogen by 50–60% and >90%, respectively [4], [12]. The other dysregulated components have not been individually suppressed or elevated in GAA^−/−^ mice, so it is not possible to predict their role in glycogen accumulation.

The large differences in GS levels between GAA^−/−^ and GAA^+/+^ mouse strains in heart, triceps, and quadriceps were not observed in all muscles. We have determined that GS levels are similar between GAA^−/−^ and wild type mice in extensor digitorum longus and soleus muscles (data not shown). We have not assessed individual fiber types for GS levels and activity. The differences observed for glycogenin between wild type and GAA^−/−^ mice were unexpected. Western blot analysis of glycogenin from GAA^−/−^ muscle lysates suggests glycogenin is modified in a way that heterogeneously increases its size.

A better understanding of the role of each of these molecular components of the glycogen pathway will aid the development of a substrate reduction therapy (SRT). The goal of SRT for Pompe disease is to reduce glycogen transport to the lysosome to a point where residual enzyme or enzyme replacement activity is sufficient to prevent pathologic accumulation. In addition to the current proposals to suppress GS [4], [9] and autophagy [12], our results suggest consideration should also be given to potential SRT therapies that might reduce GLUT4 or G6P or increase phosphorylase activity.

Administration of rhGAA to the GAA^−/−^ mice normalized not only muscle glycogen levels but also G6P, hexokinase, GS, and glycogenin levels. Thus, it would appear that correction of one aspect of the glycogen metabolic pathway (lysosomal glycogen) results in the correction of multiple other components, viz. a coordinate correction. Given the response of these other pathway components to the GAA-mediated decrease in lysosomal glycogen, it is also possible that these and other components involved in glycogen biosynthesis and degradation can be used as biomarkers to assess disease progression and response to therapies. Finally, it will be important to determine if the metabolic syndrome described here in the muscle of GAA^−/−^ mice contributes to the observed broad spectrum of disease severity in Pompe patients with the same genotype.

## Materials and Methods

### Reagents

Antibodies (GS, pGS Ser 641/645, GAPDH) were obtained from Cell Signaling (Beverly, MA, U.S.A.). Anti-glycogenin antibody was from Abnova (cat. no. H00002992-M07). The following were obtained from Sigma (St. Louis, MO) glycogen (cat. no. G-8876), glucose-1-phosphate (cat. no. G-6875), adenosine mono phosphate (cat. no. A-1752), UDP-glucose (cat. no. U-4625), glucose-6-phosphate (cat. no. G-7250), periodic acid schiff reagent. BCA kit for protein determinations and ECL supersignal detection kit were from Pierce (Rockford, Ill). UDP-[U-^14^C]-glucose (cat. no. NEC403050UC) and [U-^14^C] glucose 1-phosphate (cat. no. NEC390010UC) were from Perkin Elmer. Quick Spin Columns G-50 Sephadex were obtained from Fisher Scientific (Pittsburgh, PA). Recombinant human acid α-glucosidase (rhGAA) was obtained as previously described [13].

### Animal studies

Ethics Statement: Animal experiments were conducted in accordance with Genzyme's IACUC committee and the Guide for the Care and Use of Laboratory Animals (U.S. Department of Health and Human Services, NIH Publication no. 86-23). Genzyme's IACUC committee approved this study. Wild-type SV129 and C57BL/6 mice were obtained from The Jackson Laboratory (Bar Harbor, ME) and used as controls because the knockout mice used in this study were derived from these strains. Homozygous acid-α-glucosidase knockout mice GAA^−/−^ (referred to as Pompe mice) [14] were bred at Charles River Labs (Bedford, MA). S6K1 heterozygous knockout mice (S6K1^+/−^) on a 129/SvEv-C57BL/6 background were obtained from Taconic Farms, Inc catalog number TF0738 (Germantown, NY). S6K1^+/−^ mice were bred to homozygosity (S6K1^−/−^). S6K2 knockout mice (S6K2^−/−^) on a C57BL/6 background were obtained from Dr. George Thomas and the Friedrich Miescher Institute (Basel, Switzerland) [6]. The S6K1^−/−^ and S6K2^−/−^ mice were crossed to Pompe mice until double knockout status (GAA^−/−^ x S6K1^−/−^ and GAA^−/−^ x S6K2^−/−^) were achieved. rhGAA was dosed once per week for four weeks at 100 mg/kg intravenously. Tissues were collected one week after the last dose.

### Mouse genotyping

Mice were genotyped by polymerase chain reaction (PCR), utilizing primers designed specifically for each distinct mouse model. Determination of the S6K2 allele required two nested PCR reactions. Tissues were prepared for DNA extraction using the Qiagen QIAxtractor Robot and QIAxtractor DX reagents and plastic-ware. PCR reactions were performed using the Corbett/Qiagen CAS-1200 Robot and cycling was done using the Eppendorf Mastercycler. Post PCR products were run on the QIAxcel analyzer, fragment sizes were determined and genotypes were assigned accordingly. Primers used to discriminate alleles are as follows: S6K1^−/−^ (forward GCAGGAAGGATTCTGAAAGGA, reverse AGAAGTTGTACATTCACCATAGGAGACA, LTR reverse ATAAACCCTCTTGCAGTTGCATC),S6K2^−/−^ (step 1 forward CCATGCCTTAACCCCCTGCCTG, step 1 reverse CTGAAGGGAGGG TCCACGCGG, step 1 neo GAGCTTGGCGGCGAATGGGCTG, step 2 forward GTGCAG TTTGTCTCTGGGGATGGC, step 2 reverse GCGGCGGGCCAAGAGATCATCC, step 2 neo GGGCTGACCGCTTCCTCGTGC), GAA^−/−^ (wild type forward TGATCCATCCAAGTGCC AGG, wild type reverse primer ATCCCGCCTGTTAACCAAA, mutant forward CCTCCCACATCAGTCAAAAT, mutant reverse CCGTATCTTCCAGGGCTTAG).

### Preparation of tissue homogenates and immunoblotting

Tissue collection, protein determination by BCA, and western blotting were performed as previously described [5].

### Measurement of tissue glycogen and G6P

Tissue glycogen assays were performed as previously described [5]. G6P was determined by following the protocol provided in the G6P Assay Kit from Abcam (Cambridge, MA). Samples were deproteinized prior to performing the G6P assay, using the deproteinizing sample preparation kit from Abcam (Cambridge, MA).

### Measurement of glycogen synthase and phosphorylase activity

Glycogen synthase activity in 10–30 µg of protein lysate was determined in the presence of 4.5 mM G6P as previously described [15]. Phosphorylase activity was determined as previously described except that G-50 spin columns were used to remove unincorporated label [9], [10]. Mice were fasted overnight prior to collection of tissues.

### Measurement of glycogen synthase transcript levels

Total RNA was isolated from heart and triceps tissue using RNeasy lipid mini-kit (Qiagen, Valencia, CA). Quantitative PCR was performed using Applied Biosystem's real-time PCR 7500 system. Primer sequences for glycogen synthase were as follows: forward AGGTAGAGGTAGACGGATCAAG, reverse AGTCGTGGGTTGAAATGGAG, probe CTGCTACATAGGGAGTTCAAGGCCAA was labeled with Tam-Fam. Quantification of glycogen synthase transcript was determined relative to housekeeping ribosomal 18 s subunit transcript. The 18 s primers were obtained from Applied Biosystems (cat. no. 4319413E).

### Measurement of hexokinase activity

Hexokinase activity was determined in 10 µg of protein lysate as follows: the protein lysate was added to a reaction mixture containing 39 mM Triethanolamine pH 7.6, 0.74 mM ATP, 7.8 mM MgCl_2_, 216 mM D-glucose, 1.1 mM β-NADP, and 1 unit/ml glucose-6-phosphate-dehydrogenase (G6PD). The definition of hexokinase activity is one unit will phosphorylate 1.0 µmole of D-glucose to give glucose-6-phosphate (G6P). G6PD used the G6P to convert β-NADP to β-NADPH. The change in absorbance from the conversion of β-NADP to β-NADPH per minute was monitored at 340 nm. The millimolar extinction coefficient of β-NADPH at 340 nM is 6.22. A reaction that did not contain lysate was used as a blank and subtracted from samples containing lysate.

### Statistical analysis

Data are expressed as mean ± SEM. Data were analyzed by Student's *t* test and a one-way ANOVA with Newman-Keuls. A probability value of *P*<0.05 was considered to be statistically significant.
